# Transparency, health equity, and strategies in state-based protocols for remdesivir allocation and use

**DOI:** 10.1371/journal.pone.0257648

**Published:** 2021-10-18

**Authors:** Zippora Kiptanui, Sanchari Ghosh, Sabeen Ali, Karishma Desai, Ilene Harris

**Affiliations:** 1 Index Analytics LLC, Catonsville, MD, United States of America; 2 IMPAQ International LLC, Columbia, Maryland, United States of America; University of Utah, UNITED STATES

## Abstract

**Background:**

The Emergency Use Authorization (EUA) of remdesivir for coronavirus disease 2019 raised questions on transparency of applied strategy, and how to equitably allocate and prioritize eligible patients given limited supply of the medication. The absence of federal oversight highlighted the critical role by states in health policymaking during a pandemic.

**Objective:**

To identify public state-based protocols for remdesivir allocation and clinical guidance for prioritizing remdesivir use and assess approaches and inclusion of language promoting equitable access or mitigating health disparities.

**Methods:**

We identified remdesivir allocation strategies and clinical use guidelines for all 50 states in the U.S. and the District of Columbia accessible on state health department websites or via internet searches. Public protocols dated between May 1, 2020 and September 30, 2020 were included in the study. We reviewed strategies for allocation and clinical use, including whether protocols contained explicit language on equitable access to remdesivir or mitigating health disparities.

**Results:**

A total of 38 states had a remdesivir allocation strategy, with 33 states (87%) making these public. States used diverse allocation strategies, and only 10 (30%) of the 33 states included language on equitable allocation. A total of 30 states had remdesivir clinical use guidelines, where all were publicly accessible. All guidelines referenced recommendations by federal agencies but varied in their presentation format. Of the 30 states, 12 (40%) had guidelines that included language on equitable use. Neither an allocation strategy or clinical use guideline were identified (public or non-public) for 10 states and the District of Columbia during the study period.

**Conclusions:**

The experience with the remdesivir EUA presents an opportunity for federal and state governments to develop transparent protocols promoting fair and equal access to treatments for future pandemics.

## Introduction

During the coronavirus disease 2019 (COVID-19) pandemic, individual states have had some flexibility to customize responses for their local population. Following the Emergency Use Authorization (EUA) for remdesivir in May 2020 [1], transparency concerns about the federal government’s distribution strategy emerged [2, 3]. As such, the federal government delegated allocation responsibilities to states, where each state would develop remdesivir allocation and clinical use criteria for their communities [4, 5]. It is critical that states’ flexibility in health policymaking should not exacerbate longstanding inequitable distribution of resources and health disparities.

At the time the federal government began allocating remdesivir to states, over 50,000 Americans were hospitalized for COVID-19 [6]. With the increasing number of cases, it was evident that initial remdesivir doses available through the EUA were unlikely to meet potential demand by eligible patients. Furthermore, in August 2020, the U.S. Food and Drug Administration (FDA) expanded remdesivir’s authorization by no longer limiting use to those with severe disease. As a result, all hospitalized adult and pediatric patients “with suspected or laboratory-confirmed COVID-19” met the eligibility criteria for remdesivir [7], potentially increasing demand further. Gaps in clinical knowledge of the medication were also present, including whether remdesivir reduced mortality or which patient populations would benefit most from its use [8, 9]. The federal government did not specify criteria for initial remdesivir allocation. Disorderly allocations were evident, with remdesivir sent to some hospitals that did not need it, while other hospitals significantly impacted by COVID-19 received none [8, 10, 11].

Transparent, publicly accessible protocols are essential to reassure the public about impartiality in resource allocation [12]. Also, the disproportionate impact of COVID-19 on historically marginalized communities [13] heightened the role of clinical use guidelines. In facilities with many hospitalized patients, physicians and healthcare teams must decide which patients have priority in receiving remdesivir, raising questions about how to select patients fairly and equitably [14]. Explicit considerations of health equity in development of clinical and public health protocols have become increasingly important [15, 16]. What is unknown is the extent to which states developed transparent remdesivir-related protocols with health equity considerations once the federal government delegated this responsibility to them.

This study identified publicly available remdesivir allocation and clinical guidance from all U.S. states and the District of Columbia and assessed protocols for inclusion of language promoting equitable access or mitigating health disparities.

## Methods

### Study design

We conducted a qualitative review of health department websites for all 50 U.S. states and the District of Columbia. We identified public information dated between May 1, 2020 and September 30, 2020 on remdesivir allocation and clinical use guidance to in-state facilities. Institutional review board review was not required, as data did not involve human subjects.

Between October 8, 2020 and November 24, 2020, we searched each state’s health department website using the search term “remdesivir”. When no relevant documents were identified from state websites, the Google internet search engine was used, with these search terms: “remdesivir”, “allocation”, “administration”, “guidelines”, and the state name. Documents were included in the study if they discussed a methodology or criteria for allocating remdesivir to in-state facilities or prioritizing eligible patients within them.

Four reviewers (S.G, S.A, K.D, Z.K) collected and reviewed state remdesivir protocols. We developed review procedures to maintain consistency of review. Each reviewer was a primary reviewer for one group of states and a secondary reviewer for another–hence a robust methodology assuring reproduceable findings. Reviewers met biweekly to discuss findings and resolve disagreements.

### Data collection

For each protocol, we collected details on allocation strategy and criteria for patient use. We recorded the source (state health department website or other) and whether protocols were publicly accessible (protocol details were available without user identification or additional requests). Any documented references to recommendations from federal agencies were also collected. We identified language promoting equitable access or mitigating health disparities as explicit equity-sensitive ‘remarks’ or ‘key considerations’ statements documented in the protocols.

## Results

Among the 51 jurisdictions, 38 (75%) had a remdesivir allocation strategy and 30 (59%) had remdesivir clinical use guidelines. Thirty-three jurisdictions made the allocation strategy public, while all 30 clinical use guidelines were publicly available (**Tables 1 and S1**). Some states had a publicly available allocation strategy but no clinical use guidelines, or vice versa. Among all 51 jurisdictions, 26 (51%) developed both an allocation strategy and clinical use guidelines for remdesivir that were public. We did not find either an allocation or use protocol for 10 states and the District of Columbia. Links to the websites of the identified protocols are provided in the **S1 Table**.

**Table 1:** Summary of remdesivir allocation strategy and clinical use guidelines by state.

State	Remdesivir Allocation Strategy			Remdesivir Clinical Use Guidelines		
	Source^a^	Accessibility^b^	Equity	Source^a^	Accessibility^b^	Equity
Alabama	state	non-public	no	n/a	n/a	n/a
Alaska	n/a	n/a	n/a	n/a	n/a	n/a
Arizona	n/a	n/a	n/a	state	public	yes
Arkansas	non-state	public	no	n/a	n/a	n/a
California	state	public	yes	state	public	yes
Colorado	state	public	no	state	public	no
Connecticut	n/a	n/a	n/a	n/a	n/a	n/a
Delaware	n/a	n/a	n/a	n/a	n/a	n/a
District of Columbia	n/a	n/a	n/a	n/a	n/a	n/a
Florida	state	public	no	n/a	n/a	n/a
Georgia	state	public	no	state	public	no
Hawaii	n/a	n/a	n/a	n/a	n/a	n/a
Idaho	state	public	no	state	public	no
Illinois	state	public	yes	n/a	n/a	n/a
Indiana	non-state	public	no	n/a	n/a	n/a
Iowa	state	public	no	state	public	no
Kansas	state	public	yes	state	public	yes
Kentucky	state	public	no	state	public	no
Louisiana	state	public	no	n/a	n/a	n/a
Maine	n/a	n/a	n/a	n/a	n/a	n/a
Maryland	non-state	non-public	no	n/a	n/a	n/a
Massachusetts	non-state	non-public	no	state	public	no
Michigan	non-state	non-public	no	n/a	n/a	n/a
Minnesota	state	public	yes	state	public	yes
Mississippi	state	public	yes	state	public	yes
Missouri	state	public	no	n/a	n/a	n/a
Montana	n/a	n/a	n/a	n/a	n/a	n/a
Nebraska	state	public	no	state	public	no
Nevada	non-state	non-public	no	state	public	no
New Hampshire	state	public	no	state	public	no
New Jersey	state	public	yes	state	public	yes
New Mexico	state	public	yes	state	public	yes
New York	n/a	n/a	n/a	state	public	no
North Carolina	n/a	n/a	n/a	n/a	n/a	n/a
North Dakota	n/a	n/a	n/a	n/a	n/a	n/a
Ohio	state	public	no	state	public	yes
Oklahoma	n/a	n/a	n/a	n/a	n/a	n/a
Oregon	state	public	no	state	public	yes
Pennsylvania	state	public	yes	state	public	yes
Rhode Island	n/a	n/a	n/a	n/a	n/a	n/a
South Carolina	state	public	no	n/a	n/a	n/a
South Dakota	state	public	no	state	public	no
Tennessee	state	public	no	state	public	no
Texas	state	public	no	state	public	no
Utah	state	public	yes	state	public	yes
Vermont	state	public	no	state	public	no
Virginia	non-state	public	no	state	public	no
Washington	state	public	yes	state	public	yes
West Virginia	state	public	no	state	public	no
Wisconsin	state	public	no	state	public	no
Wyoming	state	public	no	state	public	no

Data source: State health department website or Google internet search engine (October 8 –November 24, 2020).

^a^ A “state” source was found on a state health department website; a “non-state” source was found outside of the state health department website (e.g., news article, state hospital association, health system/hospital).

^b^ A source was considered “public” if information was readily available; a source was considered “non-public” if user identification or additional requests were required to obtain information.

n/a: Information was not available publicly. States may have used private channels to distribute protocols.

### Remdesivir allocation strategies

**Table 2** presents a summary of remdesivir allocation strategies for the 33 states that made these protocols public. States used varying strategies to allocate remdesivir, ranging from no systematic procedures (allocation to facilities upon request and/or on a first come, first served basis), to those strategies that use quantitative measures of COVID-19 patient burden, including number of hospitalized COVID-19 patients, number of patients meeting EUA criteria, or a formula dependent on daily average of COVID-19 hospitalizations. Some states also used multiple strategies within their allocation protocol.

**Table 2:** Summary of remdesivir allocation strategies.

Allocation Strategy	State
By request	• Florida (as requested, no further details provided)
	• Kentucky (requests by attending physician/designee via an online form)
	• Mississippi (requested based on patients meeting EUA criteria)
	• South Carolina (online requests by licensed practitioners sent by the state’s hospital association)
	• Tennessee (requests made online for patients meeting state-specified criteria; state’s hospital association and Vanderbilt University Medical Center managed distribution)
	• Vermont (requests faxed for patients meeting state-specified criteria; distribution managed by state health department and University of Vermont Medical Center)
	• Wisconsin (requests emailed to state health department for patients meeting EUA criteria)
	• Wyoming (on request by hospitals, or by providers)
First come, first served	Kentucky
Formula (used to calculate the proportion of COVID-19 inpatients)	• Alabama (indicated a formula was used, no further details provided)
	• Iowa and Pennsylvania (total daily number of COVID-19 inpatients plus COVID-19 patients on ventilators, averaged over a 7-day period)
	• Minnesota (5-day average of non-ventilated COVID-19 inpatients)
Random allocation or lottery system	• California (random allocation among acute care hospitals)
	• Virginia (random selection based on confirmed COVID-19 patients, hospital’s ability to support remdesivir use, and remdesivir available)
Number of COVID-19 in-patients with/without FDA EUA criteria	• Colorado, New Hampshire, Utah, Washington (number of COVID-19 inpatients)
	• Indiana (first shipment to hospital with most cases)
	• Kansas (hospitals with three or more COVID-19 inpatients)
	• Louisiana (hospitals with five or more COVID-19 inpatients)
	• Nebraska (volume of COVID-19 inpatients in the prior 14 days)
	• New Mexico (inpatients on supplemental oxygen but not on high-flow oxygen, ventilator, or extracorporeal membrane oxygenation)
	• Arkansas (FDA EUA criteria)
	• Georgia (EUA criteria and hospitals with 10 or more patients on ventilator or extracorporeal membrane oxygenation)
	• South Dakota (EUA criteria and lab testing requirements remdesivir)
Number of severely ill COVID-19 patients on mechanical ventilation or extracorporeal membrane oxygenation	• Idaho, Illinois, Indiana, Missouri (hospitals with/most likely ill patients)
	• Nevada (percentage of hospital’s ICU beds compared to the state)
	• Ohio (percentage of patients on ventilators)
Geographic (based on hospital location)	• Idaho (at least one hospital in each public health district)
	• Texas (14 communities across the state)
	• West Virginia (four pre-positioned hospitals to further distribute)
Other	• Illinois (safety net hospitals, hospitals with large communities of color)
	• Maryland (based on the overall number of cases)
	• Michigan (based on the number of COVID-19-related deaths)
	• New Jersey (prioritized research participants, hospitals with clinical trials)

Of these 33 states with public protocols, 10 states (30%) had protocols with language on equitable allocation of remdesivir. In addition to considerations of COVID-19 facility burden, these protocols highlighted the need to promote equitable remdesivir allocation to facilities within the state. Illinois, for example, included non-discrimination guiding principles, specifying that allocation decisions should not be based on race, gender, or socioeconomic status.

### Remdesivir clinical use guidelines

All 30 states with guidelines for within-facility remdesivir use made these protocols publicly available. All states cited remdesivir patient use recommendations issued by the FDA, National Institutes of Health (NIH), or Centers for Disease Control and Prevention (CDC). However, as presented in **Table 3**, states varied in the format of presenting these strategies. Some states only included website links to these resources, while others explicitly stated and referenced federal recommendations, expanded on FDA EUA criteria, or delegated hospitals to develop their own protocols. Of the 30 states with public within-facility remdesivir use, 12 (40%) states had protocols that went further to include ethical standards or non-discrimination language. For instance, Minnesota developed a detailed framework which outlined different priority groups based on supply of remdesivir.

**Table 3:** Summary of strategies in remdesivir clinical use guidelines.

Guideline Strategy	State
Only provides weblinks to recommendations by federal agency	• Colorado, Massachusetts (CDC, NIH)
	• Virginia (FDA EUA, NIH)
Specific text and reference(s) to recommendations by federal agency	• California, Idaho, Iowa, Kentucky, New Mexico, New York, Oregon, Wyoming (FDA EUA, NIH)
	• Georgia, Mississippi, Nevada, New Hampshire, Ohio, Pennsylvania, Texas, Vermont, Wisconsin, (FDA EUA)
Criteria in addition to federal agency recommendations (e.g., onset or duration of symptoms, anticipated discharge date, prognosis)	• Kansas, Nebraska, New Jersey, South Dakota, Tennessee, Utah, Washington, West Virginia
	• Iowa (advisory committee recommends allocation among eligible patients to be first come, first served)
Priority groups based on specified criteria (e.g., oxygenation status, imaging, respiratory support) referencing federal agency recommendations	Minnesota, Arizona (adapted from Minnesota)
Hospitals to develop their own protocols	• Idaho, Illinois, Ohio, Pennsylvania
	• New Jersey (advisory committee recommends allocation among eligible patients to be first come, first served)

### Language on equity

We further examined the states that included health equity considerations in their remdesivir protocols. **Table 4** presents the narrative text promoting ethical and fair access to remdesivir developed by these states. While of varying length, wording on equity from these protocols clearly defined the disadvantaged population.

**Table 4:** Language on equity from remdesivir allocation strategies and/or clinical use guidelines.

State	Quotes on Ethical and/or Fair Access to Remdesivir
Arizona^a^	Based on Minnesota
California^b^	“Considerations for allocation include: a clinical prioritization team to make allocation decisions that is distinct from the clinicians providing direct care is recommended to protect the integrity of the patient-provider relationship and to ensure that decisions are fair and consistent; withholding or reserving remdesivir for future use is not recommended, particularly if there are current patients presenting with severe illness; if patients receiving remdesivir are transferred to another hospital, their remaining doses of remdesivir should transfer with them; children and pregnant mothers are still eligible to receive remdesivir through compassionate use from Gilead (instead of the donated supply); patients who have already received remdesivir should not be eligible to receive additional doses from this donated allocation."
Illinois^c^	“Remdesivir will be distributed to the hospitals that have seen the most critically ill COVID-19 patients and to safety net hospitals and hospitals treating large communities of color.”
Kansas^b^	Based on Minnesota
Minnesota^b^	• “This ethical framework for COVID-19 response is grounded in the fundamental ethical commitment that the response to a pandemic will pursue Minnesotans’ common good in ways that: are accountable, transparent, and worthy of trust; promote solidarity and mutual responsibility; respond to needs respectfully, fairly, effectively, and efficiently”
	• “To honor these fundamental value commitments, pandemic response must promote Minnesotans’ common good by balancing three ethical objectives: protect the population’s health by reducing mortality and serious morbidity; respect individuals and groups; strive for fairness and protect against systematic unfairness and inequity.”
	• “Allocation decisions should not consider or be based upon: race, ethnicity, gender, gender identity, sexual orientation or preference, religion, citizenship or immigration status, or socioeconomic status; ability to pay; age as a criterion in and of itself (this does not limit consideration of a patient’s age in clinical prognostication of likelihood to survive to hospital discharge); disability status or comorbid condition(s) as a criterion in and of itself (this does not limit consideration of a patient’s physical condition in clinical prognostication of likelihood to survive to hospital discharge); predictions about baseline life expectancy beyond the current episode of care (i.e., life expectancy if the patient were not facing the current crisis), unless the patient is imminently and irreversibly dying and/or under hospice care; first-come, first-served (should not distinguish between patients when treatment has not yet been started on equivalent patients); judgments that some people have greater “quality of life” than others; judgments that some people have greater “social value” than others.”
Mississippi^b^	“Hospitals must also agree to the following: A commitment to use the medication for appropriate patients regardless of age, sex, race, ethnicity or disability.”
New Jersey^b^	• “Promote Equitable Access: remdesivir supplies will be available at each acute care hospital in the state; this represents both the value of patients at each facility in NJ and a deterrent against privileged patients "shopping" for a facility that has this product; supply already on hand at the facility will count towards the facility’s share of the distribution.”
	• “Rights of patients admitted to general hospitals are retained, including those enumerated in N.J.S.A. 26:2H-12.8. Among these are rights pertaining to nondiscrimination, informed consent, human research, facility transfer, and confidentiality.”
New Mexico^b^	“This clinical framework is intended to provide clear parameters for the selection of patients who will receive treatment with remdesivir and reduce any potential for bias due to race, ethnicity, gender, disabilities, do not resuscitate/do not intubate (DNR/DNI) status, socioeconomic status, rural residency, age, education status, or occupation.”
Ohio^a^	“In all cases, the hospital will ensure that the allocation process does not use discriminatory criteria such as disability status, race or ethnicity, sex, sexual orientation, gender, religion, socioeconomic status, housing status, incarceration status, etc.”
Oregon^a^	• “The distribution and use of the investigational drug remdesivir must take into consideration the historical consequences for people of color and individuals with disabilities who have been impacted by lack of disclosure related to experimental drugs, procedures and testing. The distribution and use of remdesivir must include a focus on health equity and take current and historical experiences into consideration.”
	• “Oregon Health Authority’s definition of health equity: Oregon will have established a health system that creates health equity when all people can reach their full health potential and well-being and are not disadvantaged by their race, ethnicity, language, disability, gender, gender identity, sexual orientation, social class, intersections among these communities or identities, or other socially determined circumstances. Achieving health equity requires the ongoing collaboration of all regions and sectors of the state, including tribal governments to address: the equitable distribution or redistributing of resources and power; and recognizing, reconciling and rectifying historical and contemporary injustices.”
	• “Treatment decisions should NOT consider or be based upon: race, ethnicity, gender, gender identity, sexual orientation or preference, religion, citizenship or immigration status, or socioeconomic status; ability to pay; age as a criterion in and of itself; disability status or comorbid condition(s) as a criterion in and of itself; predictions about baseline life expectancy beyond the current episode of care (i.e., life expectancy if the patient were not facing the current crisis); judgments that some people have greater “quality of life” than others; judgments that some people have greater “social value” than others.”
Pennsylvania^b^	“Consistent with accepted standards during public health emergencies, and the Interim Pennsylvania Crisis Standards of Care for Pandemic Guidelines, this framework is designed to achieve the following ethical goals: to safeguard the public’s health by allocating scarce treatments to maximize community benefit; to create meaningful access for all patients, all patients who meet clinical eligibility criteria should have a chance to receive treatment; to ensure that no one is excluded from access based on age, disability, religion, race, ethnicity, national origin, immigration status, gender, sexual orientation, or gender identity and to ensure that no one is denied access based on stereotypes, perceived quality of life, or judgements about a person’s worth; to ensure that all patients receive individualized assessments by clinicians, based on the best available objective medical evidence; to proactively mitigate health disparities in COVID-19 outcomes.”
Utah^b^	“This protocol does not discriminate based on race, color, national origin, disability, sex, or exercise of conscience and religion. It meets the CSC ethical goals of fairness, duty to care, transparency, consistency, proportionality, and accountability.”
Washington^b^	• “Non-Discrimination Statement: In applying this distribution plan, health programs or activities that receive federal financial assistance agree with Section 1557 of the Affordable Care Act and Section 504 of the Rehabilitation Act which “prohibit discrimination on the basis of race, color, national origin, disability, age, sex, and exercise of conscience and religion in HHS-funded programs.” The federal Office of Civil Rights has issued recent guidance that emphasizes that “persons with disabilities should not be denied medical care on the basis of stereotypes, assessments of quality of life, or judgments about a person’s relative ‘worth’ based on the presence or absence of disabilities. Decisions by health programs or activities that receive federal financial assistance concerning whether an individual is a candidate for treatment should be based on an individualized assessment of the patient based on the best available objective medical evidence.”
	• Contains the National Academy of Medicine ethical principles: fairness, duty to care, duty to steward resources, transparency, consistency, proportionality, accountability
	• “Allocation decisions should not be based upon a patient’s” race, ethnicity, language, sex, gender identity, sexual orientation, religion or spiritual beliefs, disability status, citizenship or immigration status, or socioeconomic status; ability to pay; age as a criterion in and of itself; disability status or comorbid condition(s) as a criterion in and of itself; predictions about baseline life expectancy beyond the current episode of care (i.e., life expectancy if the patient were not facing the current crisis), unless the patient is imminently and irreversibly dying or terminally ill with life expectancy under 6 months (e.g., eligible for admission to hospice); judgments that some people have greater “quality of life” than others; judgments that some people have greater “social value” than others.”

^a^In clinical use guidelines.

^b^In both allocation strategy and clinical use guidelines.

^c^In allocation strategy.

## Discussion

Despite the limited federal oversight, our review found that 33 states had a remdesivir allocation strategy and 30 states had remdesivir clinical use guidelines that were publicly accessible. However, the strategies for allocation and clinical use in these protocols varied, and fewer than half of these states included recommendations that promote fair and equal access to remdesivir.

States with public protocols used diverse strategies to allocate remdesivir to in-state facilities, potentially a reflection of ongoing debate over how best to allocate this medication [8, 9]. While we presently lack consensus on the most effective approach for allocating scarce medications during a pandemic, current research shows that COVID-19 has disproportionately affected racial and ethnic minority communities [17, 18]. Thus, the absence of language acknowledging and mitigating this disparity in a majority of the allocation protocols was concerning, particularly when employing these explicit policy statements that draw awareness and promote conscious efforts is a key characteristic of improving equity in health care systems [19]. Only 10 of 33 states (30%) with public remdesivir allocation strategies had considerations for ethical allocation approaches. Given the current disparities in COVID-19 burden of illness, regardless of the chosen allocation strategy, an ethical allocation approach for remdesivir should prioritize these communities with the greatest disease burden. Well-crafted, clear clinical guidelines promote quality of care by reducing health care variations and dissuading potentially harmful interventions [20].

All public protocols assessed cited remdesivir patient use recommendations by federal agencies but varied in their presentation format, possibly highlighting the current lack of shared methodology for guideline development [21]. Creating clinical use guidelines for a federally restricted medication would not be expected to fall within the responsibilities of the state; however, state governments should be expected to promote a standard approach for a scarce resource they are allocating. Ideally, access to remdesivir should be based on a patient’s clinical factors, as well as the likelihood and magnitude of benefit from the medication, and not based on race, age, gender, or other socioeconomic attributes. While a majority of the 30 states with public remdesivir clinical use guidelines provided specific patient use criteria, only 12 (40%) states included additional ethical standards or non-discrimination language. Guidelines that are evidence-based, avoid discrimination, and mitigate health disparities could help physicians make these difficult decisions [22].

The COVID-19 pandemic is not the first time the U.S. has faced the issue of allocating scarce resources during a public health emergency. Following issuance of an EUA for peramivir during the 2009 H1N1 influenza pandemic, the CDC managed the drug’s distribution directly to hospitals [23]. The CDC promptly provided an online portal for physicians to request peramivir for individual patients, where criteria for approval included suspected or confirmed H1N1 virus infection, and either lack of response or inability to receive oral or inhaled antiviral treatment [24]. Once approved, the medication was sent to the requesting clinician within 24 hours. The peramivir EUA provides an example of how a single designated federal agency can successfully distribute scarce resources through a transparent and clearly defined process [25]. It is important to note that the H1N1 influenza pandemic was of moderate scale compared to that from COVID-19, and other treatments were available for use in influenza. The question still remains whether the federal government could have done more to promote a cohesive response to ensure equitable remdesivir allocation across states. Multiple frameworks that include fair and equitable allocation of scarce resources in a pandemic already exist and could be customized by governments for the allocation of pharmaceutical countermeasures. In their *Crisis Standards of Care*, the Office of the Assistant Secretary for Preparedness and Response recommends an allocation process that is “ethically defensible, fair, and transparent” [26]. Similarly, a multidisciplinary team developed the University of Pittsburgh Guidance Model, combines clinical and ethical criteria to allocate scarce resources and to mitigate the impact of social inequities [27]. Emanuel et al. provide six ethical principle recommendations for fair resource allocation during COVID-19 [28]. A federal framework allowing for adaptation to local state context could guide allocation and use of scarce resources. Unfortunately, the process for remdesivir allocation made it difficult for the federal government to redistribute allocations based on the rapidly changing COVID-19 burden across different parts of the country. Responsibility was given to states to determine reallocation of excess remdesivir to other hospitals within their jurisdictions, or to quickly notify the federal government to reallocate their product to other states [5].

We did not find remdesivir allocation strategies or clinical use guidelines for 10 states and the District of Columbia. Lack of transparency on how scarce resources are allocated adds to the lack of public trust in allocation policies [29, 30]. Transparency also allows for modeling best practices and serving as a resource to other states; Minnesota’s remdesivir protocols were cited as a resource to create protocols in Arizona and Kansas. It is also possible that some states did not need these protocols. Oregon, for example, did not develop one and documented that the remdesivir supply was sufficient to meet the demand from hospitalized COVID-19 patients in the state. However, documenting these protocols *a priori* could alleviate potential triage dilemmas posed by the pandemic.

For future situations that pose a similar threat as the COVID-19 pandemic, the federal government could recommend specific evidence-based requirements for states to address in their allocation guidelines for hospitals, such as addressing how to ensure an equitable and transparent distribution process both within the state and within healthcare facilities [2, 31].

### Limitations

There were information gaps for some states. We did not find public protocols on remdesivir allocation or use from 10 states and the District of Columbia. Some states had public protocols on remdesivir allocation but not on patient use, or vice versa. While we chose to focus on information which was publicly available to reflect transparency, it is possible that these documents were available in non-public ways. States may have used other channels to distribute their protocols, such as via email, or in the form of a hard copy. Despite these gaps, the majority of U.S. jurisdictions were included in the study– 63% for remdesivir allocation review and 59% for remdesivir clinical use review. Another study limitation is that we focused on state-level guidance only; there may be other facility-specific protocols that have a greater influence on patient access. Notably, a survey of 66 facilities across 28 states conducted during our study period found that remdesivir use guidelines were inconsistent and did not include ethical or equitable standards [14]. Our study findings are also limited to the data collection period; it is possible that some states may have since updated these protocols. Additionally, we based our definition of equitable access on the inclusion of explicit language promoting fair allocation in state protocols. However, our study did not further explore whether states or hospitals executed and enforced equitable allocations or if strategies were deemed equitable without explicit language. Further research can examine the different strategies to improve our understanding on fairness.

## Conclusion

This review highlights the variation in strategy and, in some cases, the absence of publicly accessible state guidance for remdesivir allocation and use. Fewer than half of the states reviewed had transparent remdesivir allocation and/or clinical use protocols to guide fair and equitable access. Future research could assess the effectiveness of various strategies and models used by the states, focusing on whether equitable allocation of remdesivir was achieved. This experience with the remdesivir EUA also presents an opportunity for the federal and state governments to develop allocation and clinical use protocols promoting fair and equal access to treatments for future pandemics. Notably, both federal and state governments have issued protocols to ensure equity in distribution of COVID-19 vaccines. Further work could also assess whether these vaccination strategies were effective.

## Supporting information

S1 Table Source of information for remdesivir allocation strategies and clinical use guidelines. (ZIP)
