# Supplementary figures and images for: Transparency, health equity, and strategies in state-based protocols for remdesivir allocation and use
Source: PLoS One. 2021 Oct 18;16(10):e0257648. doi: 10.1371/journal.pone.0257648 (PMC8523064; doi:10.1371/journal.pone.0257648)

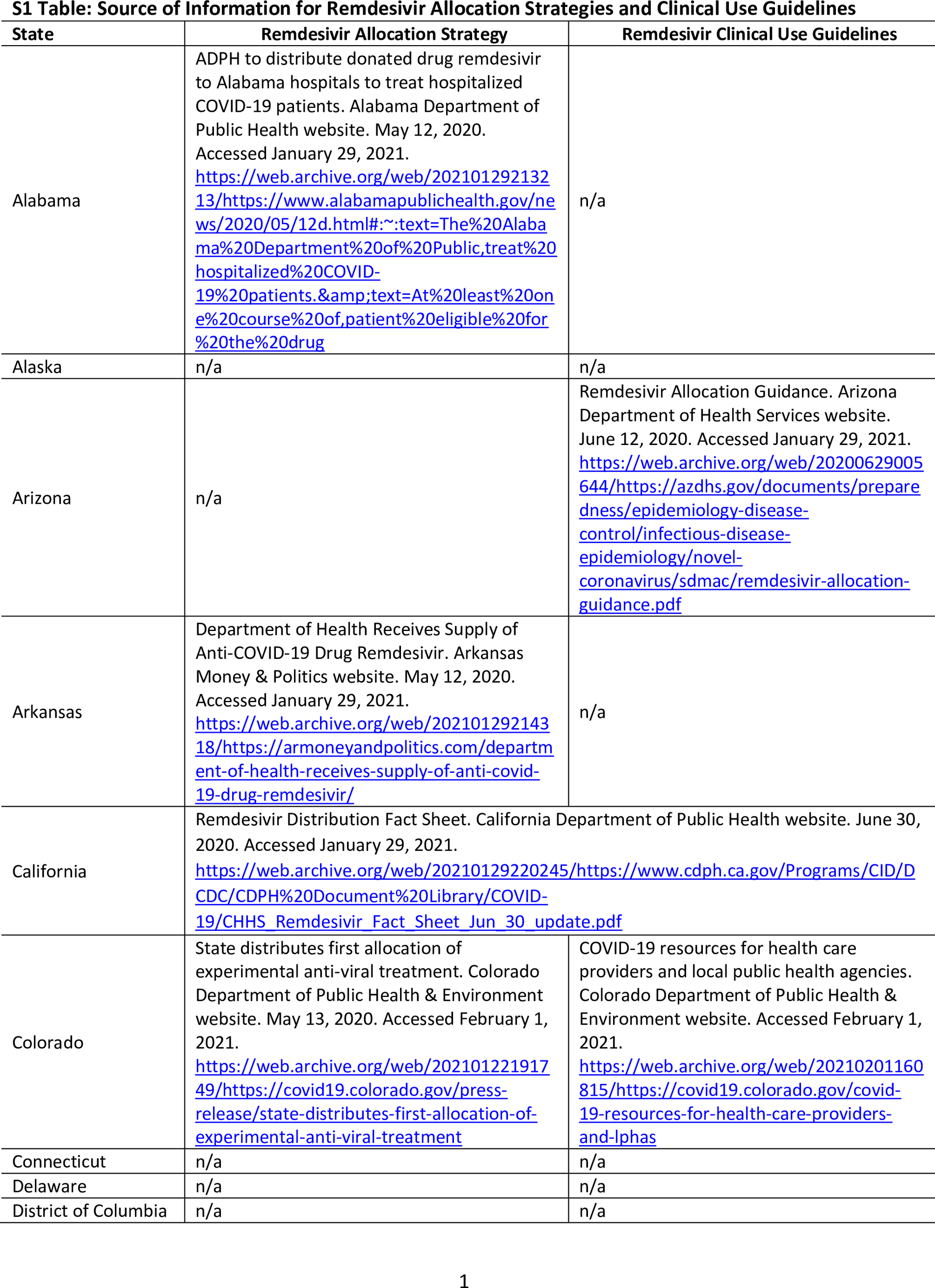

Supplement: S1 Table — (ZIP) [file pone.0257648.s001.zip › PACE Corrected/Supporting Information (1).tif]

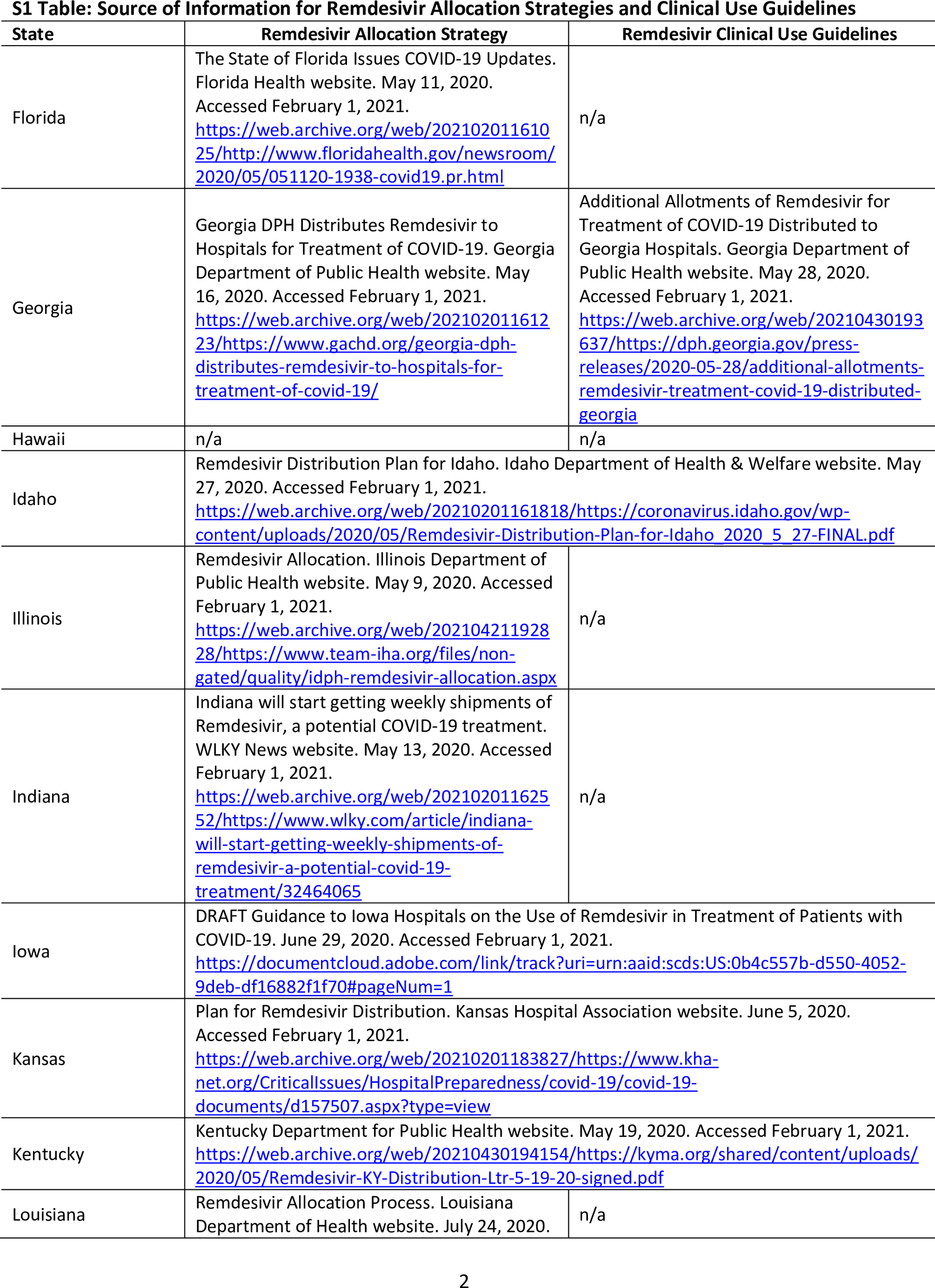

Supplement: S1 Table — (ZIP) [file pone.0257648.s001.zip › PACE Corrected/Supporting Information (1).tif]

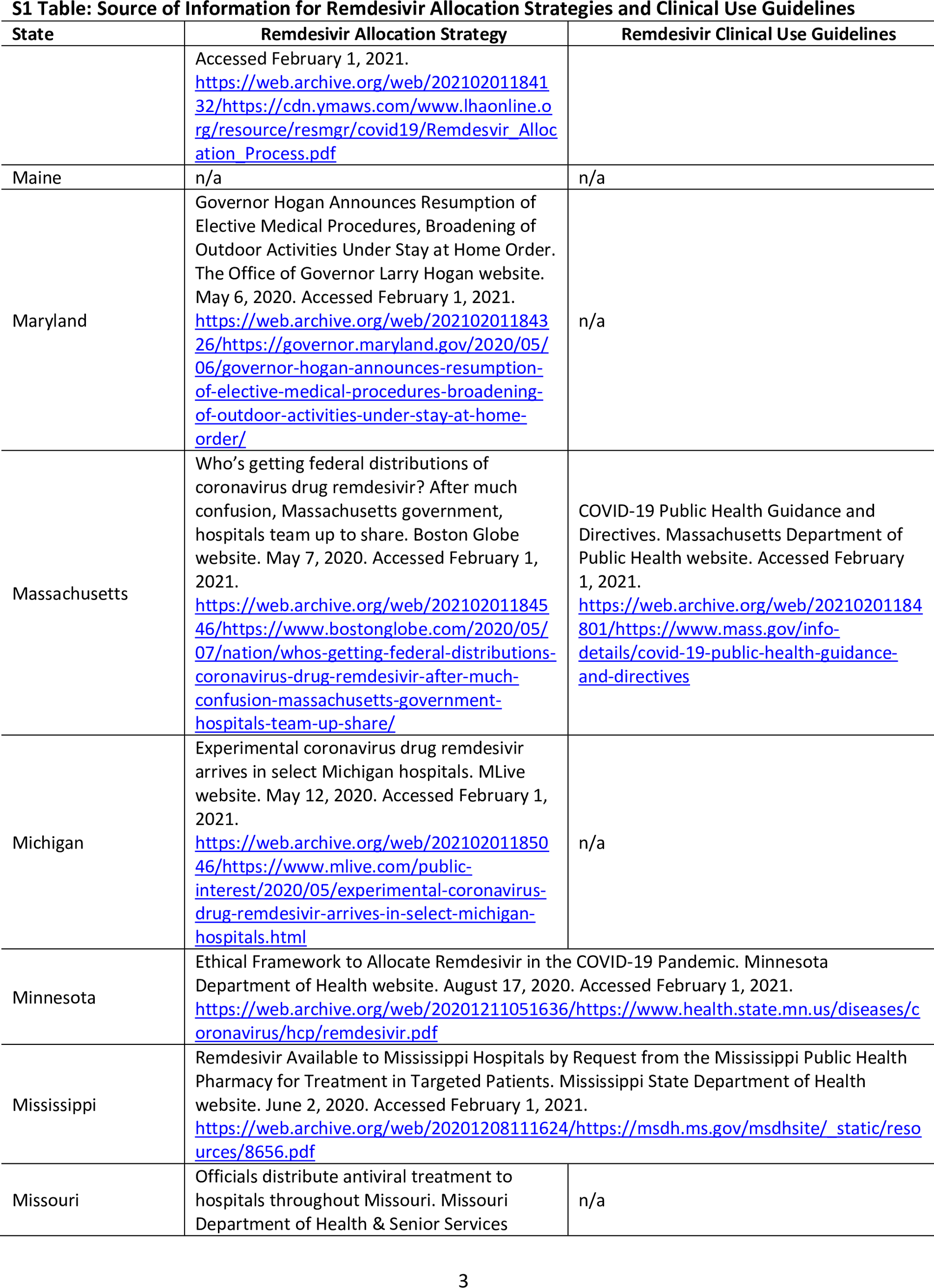

Supplement: S1 Table — (ZIP) [file pone.0257648.s001.zip › PACE Corrected/Supporting Information (1).tif]

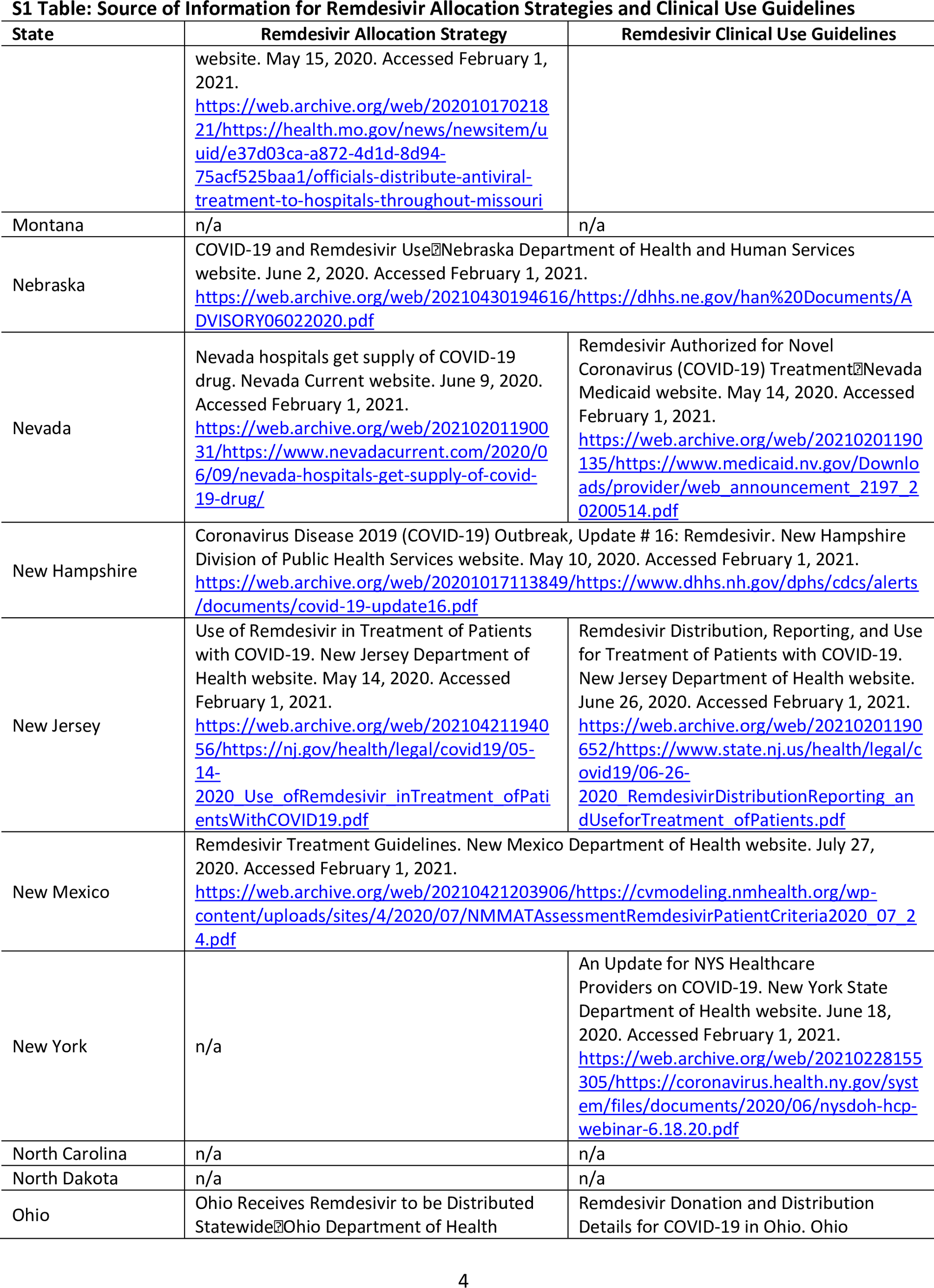

Supplement: S1 Table — (ZIP) [file pone.0257648.s001.zip › PACE Corrected/Supporting Information (1).tif]

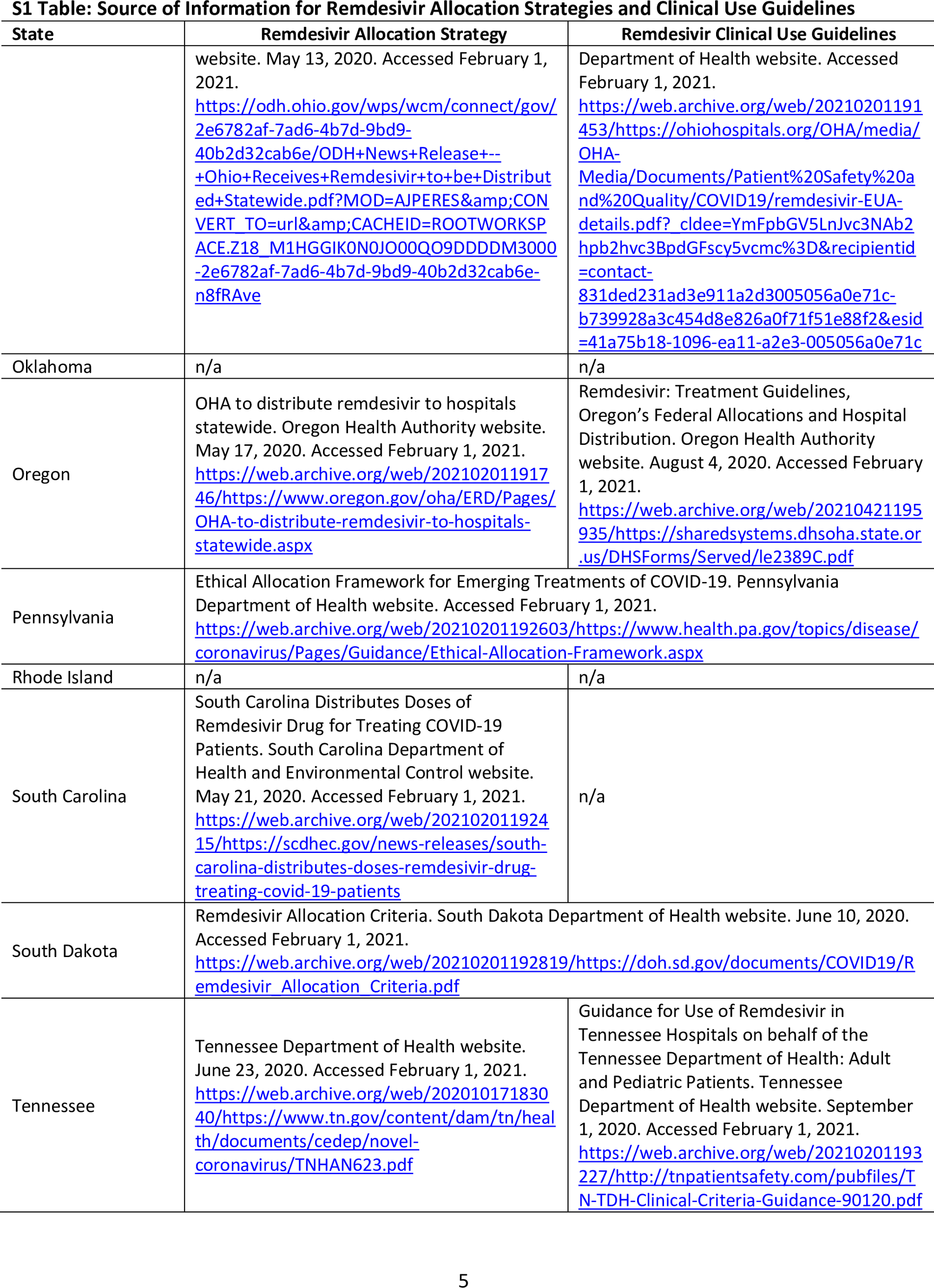

Supplement: S1 Table — (ZIP) [file pone.0257648.s001.zip › PACE Corrected/Supporting Information (1).tif]

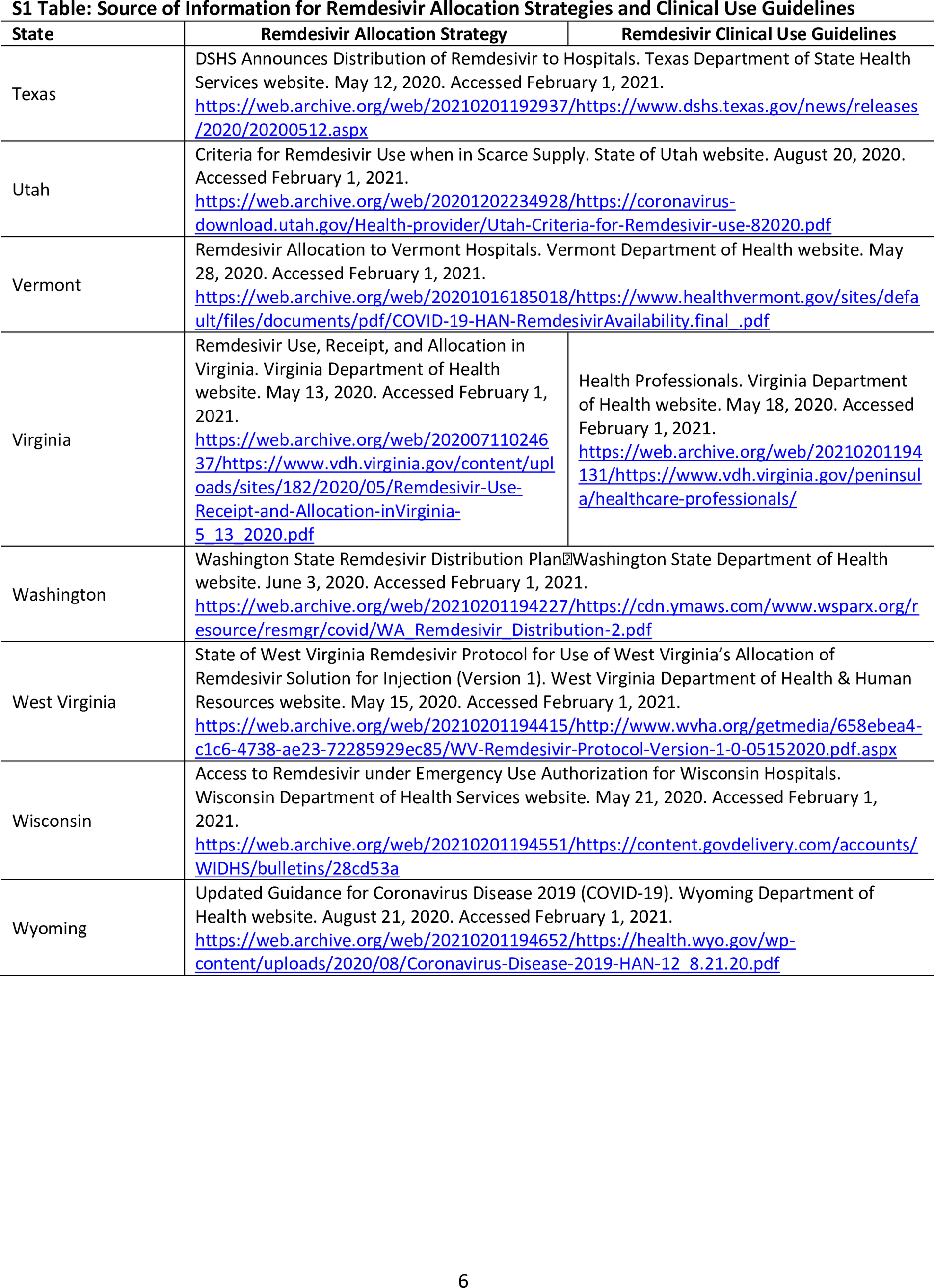

Supplement: S1 Table — (ZIP) [file pone.0257648.s001.zip › PACE Corrected/Supporting Information (1).tif]
